# Associations of Physical Activity, Screen Time, and Adiposity with Elevated Blood Pressure in Preschool Children

**DOI:** 10.3390/healthcare14213821

**Published:** 2026-11-09

**Authors:** Jarosław Herbert, Vasylyna Petrovets, Weronika Pigóra, Aleksandra Wiazicka, Krzysztof Warchoł, Piotr Matłosz

**Affiliations:** Faculty of Physical Culture Sciences, Collegium Medicum, University of Rzeszów, 35-959 Rzeszów, Poland; vpetrovets@ur.edu.pl (V.P.); wp120275@stud.ur.edu.pl (W.P.); aw120318@stud.ur.edu.pl (A.W.); kwarchol@ur.edu.pl (K.W.); pmatlosz@ur.edu.pl (P.M.)

**Keywords:** preschool children, elevated blood pressure, adiposity, physical activity

## Abstract

Background/Objectives: Elevated screening blood pressure (BP) in preschool children is an important cardiovascular health concern. This cross-sectional study examined associations between objectively measured moderate-to-vigorous physical activity (MVPA), parent-reported screen time, adiposity indicators, and screening BP among children aged 5–6 years in Rzeszów, Poland. Methods: The study included 128 children attending public kindergartens. BP was assessed during a single screening session using an oscillometric device. Adiposity was evaluated using body mass index (BMI), bioelectrical impedance-derived body fat percentage (FAT), and waist-to-height ratio (WHtR). Physical activity was monitored using ActiGraph accelerometers for seven consecutive days, with valid accelerometry data available for 81 children. Screen time was parent-reported. Associations were examined using logistic regression and modified Poisson regression with robust variance estimation. Results: Screening SBP (systolic blood pressure) and/or DBP (diastolic blood pressure) ≥ 95th percentile was observed in 74 children (57.8%). FAT ≥ 85th percentile was significantly associated with elevated screening BP in adjusted logistic regression (aOR = 2.47; 95% CI: 1.09–5.59) and modified Poisson regression (aPR = 1.41; 95% CI: 1.04–1.92). BMI ≥ 85th percentile and WHtR ≥ 0.50 were not significantly associated with screening BP in the adjusted models. Weekend screen time > 1 h/day was associated with higher odds of elevated screening BP in adjusted logistic models, whereas no statistically significant association was observed for MVPA. Conclusions: BIA-derived body fat percentage was associated with elevated screening BP, but these findings do not establish its superiority over other adiposity indicators. The unusually high proportion of elevated BP values and the single-session screening assessment warrant caution. Larger prospective studies with repeated BP measurements are needed to confirm these associations.

## 1. Introduction

Preschool age is an important period for the development of factors related to later cardiovascular health, including blood pressure (BP). The ENVIRONAGE cohort study showed that higher BP levels observed as early as this stage of life are associated with a greater likelihood of persistently elevated BP later in childhood [1]. In recent years, elevated BP has been increasingly recognized in children, largely in parallel with the rising prevalence of overweight and obesity [2,3]. Accordingly, the American Academy of Pediatrics guidelines emphasize the importance of early detection of elevated BP and the identification of children at increased risk [4].

Excess adiposity is considered one of the most important modifiable risk factors for elevated BP in children. However, assessment based solely on body mass index (BMI) may not fully reflect the degree of adiposity, body fat distribution, or the associated cardiometabolic risk. Therefore, additional measures, such as body fat percentage (FAT) and indices of central adiposity, including the waist-to-height ratio (WHtR), may provide clinically relevant information. Jeong and Kim [5] confirmed a strong association between excess body weight and hypertension in pediatric populations, whereas Tang et al. [6] demonstrated that, among children aged 2–6 years, indices of central adiposity may be more strongly associated with elevated BP than BMI.

Physical activity and sedentary behaviors, including screen time, are modifiable health behaviors that are important for cardiovascular health in children. Higher levels of moderate-to-vigorous physical activity (MVPA) are associated with a more favorable cardiometabolic risk profile, and interventions aimed at increasing physical activity may reduce BP levels, particularly among children with overweight or obesity [7,8,9]. In contrast, longer screen time has been associated with a higher risk of hypertension and a less favorable cardiometabolic profile in children [10,11].

Although the importance of adiposity, physical activity, and screen time for cardiovascular health in children is well established, most previous studies have focused on school-aged children or examined these factors separately. Relatively few studies have simultaneously investigated accelerometer-measured MVPA, parent-reported screen time, and multiple indicators of adiposity in relation to elevated BP, particularly among preschool-aged children.

Therefore, the aim of the present study was to determine the associations between objectively measured moderate-to-vigorous physical activity, screen time, adiposity indicators, and elevated BP among children aged 5–6 years. We hypothesized that greater adiposity, lower MVPA levels, and longer screen time would be associated with elevated BP.

## 2. Materials and Methods

### 2.1. Study Design and Study Sample

A cross-sectional study was conducted in October and November 2025 among children aged 5–6 years attending public kindergartens in Rzeszów, Poland. A multistage sampling procedure was employed. The city was divided into five geographical areas, from each of which three kindergartens were randomly selected. In total, 15 of the 30 public kindergartens in Rzeszów were included in the study.

A total of 200 children aged 5–6 years were invited to participate. Written informed consent was obtained from the parents or legal guardians of 175 children (87.5% of those invited), whereas consent was not obtained for 25 children. Of the 175 children whose parents or legal guardians initially provided written informed consent, 47 were not included in the final analytical sample, primarily due to subsequent withdrawal of parental consent or non-completion of the required measurements and assessments. The final study sample comprised 128 children, representing 64.0% of all those invited to participate.

Valid blood pressure measurements and anthropometric data were obtained for all 128 participants. Complete screen time data for weekdays and weekends were available for 122 and 123 children, respectively, while valid accelerometry data were obtained from 81 participants. Differences in sample sizes across analyses were attributable to data availability.

**Figure 1:**
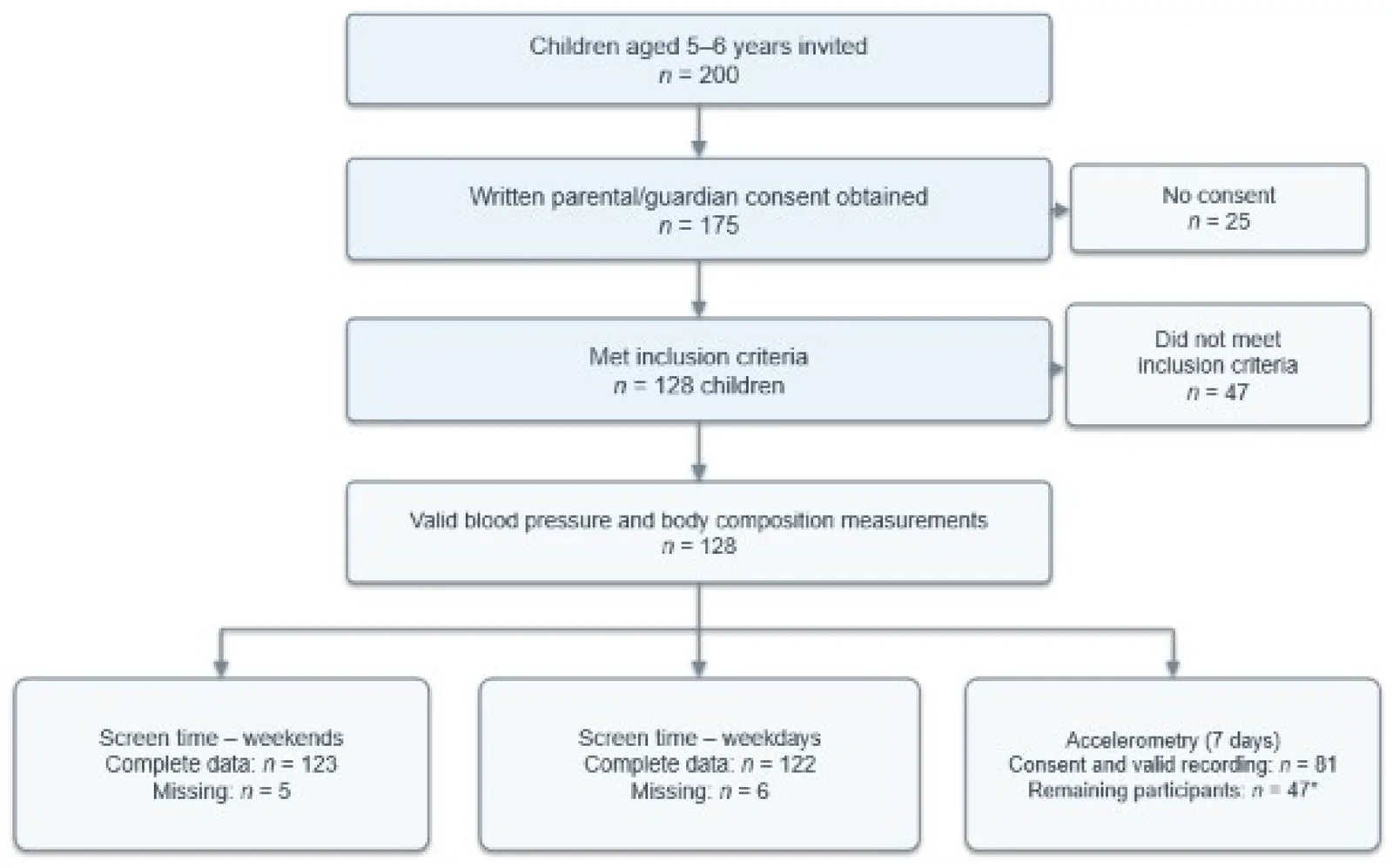
Participant recruitment and data availability across analyses. * For the remaining 47 participants, lack of consent for accelerometry was not distinguished from an invalid recording. Of the 200 children invited, 72 were not included in the final sample: 25 without consent and 47 who did not meet the inclusion criteria.

The random selection of kindergartens ensured geographical diversity in the locations of the participating institutions. However, because children’s participation was voluntary and required parental or legal guardian consent, the study sample cannot be considered representative of the entire population of preschool-aged children in Rzeszów. The final study sample comprised 128 preschool children, including 61 five-year-olds and 67 six-year-olds. Of these, 65 participants were girls (50.8%) and 63 were boys (49.2%) (Figure 1).

The inclusion criteria were: age 5–6 years, attendance at a public kindergarten in Rzeszów, and written informed consent provided by a parent or legal guardian. The exclusion criteria were: contraindications to body composition assessment (e.g., the presence of a pacemaker or other electronic implants), mental and/or physical disabilities preventing completion of the study procedures, withdrawal of parental consent, or failure to participate in the required measurements and assessments.

### 2.2. Measurements

#### 2.2.1. Blood Pressure Measurements

Blood pressure (BP) was measured using an automated oscillometric device (Omron HBP-1100; Omron Healthcare Co., Ltd., Kyoto, Japan), following a standardized protocol based on the recommendations of the European Society of Hypertension (ESH). Measurements were performed in a quiet environment after the children had rested in a seated position for at least 5 min. During the measurements, the right arm was supported at heart level, and both feet were resting flat on the floor. Children avoided vigorous physical activity immediately before the assessment. Cuffs were individually selected according to each child’s arm circumference, using cuff sizes appropriate for arm circumference ranges of 12–18 cm and 17–22 cm.

Three consecutive measurements of systolic blood pressure (SBP) and diastolic blood pressure (DBP) were obtained from the right arm of each participant, with 1-min intervals between measurements. The mean of the second and third measurements was used for statistical analyses. All measurements were performed using the oscillometric method, without additional confirmation by auscultation.

SBP and DBP were classified according to the reference values presented in Tables 2 and 3 of the 2016 ESH guidelines [12], which provide sex-specific blood pressure percentiles based on age and height. Height percentiles were determined using the Polish growth reference standards published by Kułaga et al. [13]. SBP and DBP values below the 90th percentile were classified as normal, values from the 90th to below the 95th percentile as high-normal, and values at or above the 95th percentile (≥95th percentile) as elevated. A child was classified as having BP ≥ 95th percentile if the mean SBP and/or DBP was equal to or exceeded the corresponding 95th percentile threshold. This definition encompassed isolated systolic elevation, isolated diastolic elevation, and concurrent elevation of both parameters.

Because blood pressure measurements were obtained during a single assessment session, values at or above the 95th percentile were interpreted as screening findings indicating the need for further clinical evaluation rather than as a basis for diagnosing hypertension.

#### 2.2.2. Anthropometric Measurements

Anthropometric measurements were taken between 8:00 a.m. and 10:00 a.m. by experienced researchers (ISAK—International Society for the Advancement of Kinanthropometry Level 1 and Level 2-). Body height and waist circumference were measured using a Seca 213 stadiometer (Seca GmbH & Co. KG, Hamburg, Germany) and a Lufkin flexible steel anthropometric tape (Apex Tool Group, Sparks, MD, USA), respectively, to the nearest 0.1 cm. Body weight and body fat percentage (FAT) were assessed using bioelectrical impedance analysis (BIA) with a Tanita DC-430 S body composition analyzer (Tanita Corporation, Tokyo, Japan). According to the manufacturer’s specifications, the device supports body composition assessment in children aged 5 years and older.

Measurements were performed between 8:00 and 10:00 a.m., with children wearing light clothing and no shoes or socks and standing barefoot on the device’s electrodes. Before the assessment, parents or legal guardians were instructed to ensure that their children fasted for at least 8 h prior to measurement.

FAT values were automatically estimated by the analyzer’s software using the manufacturer’s proprietary prediction algorithm. No additional external prediction equation was applied.

Elevated body fat percentage was defined as FAT ≥ 85th percentile, based on the sex-specific reference curves developed by McCarthy et al. (2006) for children and adolescents aged 5–18 years [14].

Body mass index (BMI) was calculated as body weight in kilograms divided by height in meters squared (kg/m^2^), while waist-to-height ratio (WHtR) was calculated as waist circumference divided by height. Excess weight was defined as BMI ≥ 85th percentile using Polish age- and sex-specific reference values for preschool children [13]. WHtR ≥ 0.50 was classified as elevated.

#### 2.2.3. Physical Activity Measurements

Physical activity was assessed using an ActiGraph wGT3X-BT accelerometer (ActiGraph, Pensacola, FA, USA) worn on the right hip. Data were collected continuously for 7 consecutive calendar days, beginning on the day of anthropometric measurements, at a sampling rate of 30 Hz [15]. Waking wear-time data were reintegrated into 5-s epochs. A day was considered valid when it contained at least 600 min of waking wear time, and participants were included in the accelerometry analyses when they had at least four valid days, including a minimum of three weekdays and one weekend day [16]. Mean daily time spent in moderate-to-vigorous physical activity (MVPA; min/day) was calculated across all valid days. Activity intensity was classified using the Evenson et al. [17] cut-points, corresponding to ≥2296 counts/min for MVPA (equivalent to ≥192 counts/5-s epoch). ActiLife 6.13 software was used for data processing. In accordance with the 2020 WHO (World Health Organization) guidelines for children and adolescents aged 5–17 years, participants accumulating an average of ≥60 min/day of MVPA were categorized as meeting the recommendation, whereas those accumulating <60 min/day were categorized as below the recommendation [18].

A parent-reported questionnaire was used as a supplementary research tool to collect basic demographic information and children’s screen time.

Children wore the accelerometers continuously for 24 h per day, including during sleep. Parents or legal guardians were instructed to remove the devices only during water-based activities, such as bathing, showering, or swimming. Non-wear periods were identified using the Choi algorithm [19], whereas sleep and waking periods were identified using the Sadeh algorithm [20]. Both sleep and non-wear periods were excluded from the analyses of physical activity and sedentary behavior. The Sadeh algorithm was used exclusively to identify waking periods, as sleep parameters were not analyzed in the present study. Parents did not complete accelerometer wear-time diaries or physical activity diaries; non-wear and sleep–wake periods were determined from the recorded accelerometer data using the specified algorithms.

An author-developed, parent-reported questionnaire was used as a supplementary research tool to collect basic demographic information and children’s screen time. The assessment followed the methodology used in previous research by our team involving preschool-aged children [21].

Screen time was defined as time spent watching television or using smartphones, computers, and video games. Parents or legal guardians estimated their children’s average daily screen time separately for weekdays and weekend days. For analytical purposes, screen time was categorized as ≤1 h/day and >1 h/day, using the threshold summarized by McArthur et al. [22].

The questionnaire was not independently validated. Therefore, the reported screen time was considered a subjective parental estimate rather than an objectively measured indicator of sedentary behavior. Written informed consent was obtained from parents or legal guardians, who were also informed about the study procedures.

### 2.3. Ethics

Participation in the study was voluntary. The study was conducted in accordance with the Declaration of Helsinki and under the ethical approval granted by the Bioethics Committee of the University of Rzeszów, Poland (Resolution No. 14/10/2018, dated 11 October 2018).

Recruitment and data collection were conducted in October–November 2025 within the framework of the research project originally approved in 2018. The project documentation was subsequently updated under Resolution No. 17/01/2026/W, dated 30 January 2026, and Resolution No. 43/03/2026, dated 27 March 2026. These updates concerned research documentation and did not involve changes to the measurement procedures used during data collection in 2025.

Written informed consent was obtained from parents or legal guardians of all participants before their inclusion in the study.

### 2.4. Statistical Analysis

Statistical analyses were performed using Statistica 13.3 and MedCalc version 22. The Shapiro–Wilk test was used to assess the normality of continuous variable distributions. Depending on the analysis, continuous variables were summarized as mean ± standard deviation (SD), median with quartile deviation (Me ± QD), or median with interquartile range (Q1–Q3). Categorical variables were presented as absolute numbers and percentages.

Between-group differences were evaluated using the Mann–Whitney U test for non-parametric comparisons. For comparisons of BMI, body fat percentage (FAT), waist-to-height ratio (WHtR), and moderate-to-vigorous physical activity (MVPA) between screening blood pressure (BP) categories presented in Table 2, Welch’s independent-samples *t*-test was used. Differences in the distribution of dichotomous anthropometric and MVPA categories by sex were examined using Fisher’s exact test. Associations between continuous variables and systolic blood pressure (SBP) or diastolic blood pressure (DBP) were evaluated using Spearman’s rank correlation coefficients (r). Correlation analyses were conducted for the total sample and separately for girls and boys, using available paired observations.

Unadjusted odds ratios (ORs) with 95% confidence intervals (CIs) were calculated to examine associations between screening BP ≥ 95th percentile and BMI ≥ 85th percentile, FAT ≥ 85th percentile, WHtR ≥ 0.50, MVPA < 60 min/day, weekday screen time > 1 h/day, and weekend screen time > 1 h/day.

Three separate multivariable logistic regression models were fitted to investigate associations between adiposity indicators and screening BP ≥ 95th percentile. Each model included one adiposity indicator (BMI, FAT, or WHtR) and was adjusted for age, sex, weekday screen time, and weekend screen time. Adjusted odds ratios (aORs) and their 95% CIs were reported. The adiposity indicators were examined in separate models, and no formal comparison of their predictive performance was performed.

Given the high proportion of children classified as having screening BP ≥ 95th percentile, modified Poisson regression with robust (sandwich) variance estimation was additionally used to estimate adjusted prevalence ratios (aPRs) and their 95% CIs. Three separate models were fitted for BMI, FAT, and WHtR, respectively, using the same adjustment covariates as the multivariable logistic regression models.

To assess the potential influence of clustering within kindergartens, mixed-effects logistic regression models with a random intercept for kindergarten were fitted as a sensitivity analysis. Each model included one adiposity indicator and was adjusted for age, sex, weekday screen time, and weekend screen time. The intraclass correlation coefficient (ICC) was estimated from the random-intercept variance.

Analyses were conducted using available-case data, and no imputation of missing values was performed. The primary multivariable models included participants with complete information on BP, adiposity indicators, age, sex, and weekday and weekend screen time (*n* = 122). Because valid accelerometry data were available for only 81 of the 128 participants, MVPA was examined in separate analyses and was not included in the primary multivariable models to avoid a substantial reduction in sample size.

Sex-stratified analyses were considered exploratory. No formal interaction tests between sex and screen time were performed; therefore, differences in statistical significance between girls and boys were not interpreted as evidence of sex-specific associations. No formal correction for multiple comparisons was applied, and results from exploratory analyses were interpreted with caution. Statistical significance was set at *p* < 0.05.

## 3. Results

Following reassessment of individual blood pressure classifications using the 2016 ESH reference tables, 74 of the 128 participants (57.8%) were classified as having SBP and/or DBP ≥ 95th percentile, whereas 54 children (42.2%) had both parameters below the 95th percentile. The mean SBP was 112.68 ± 12.21 mmHg, and the mean DBP was 70.84 ± 10.81 mmHg. Median SBP was 110.25 mmHg (Q1–Q3: 104.88–119.00 mmHg), whereas median DBP was 69.50 mmHg (Q1–Q3: 63.38–76.13 mmHg).

Isolated systolic elevation was observed in 25 children (19.5%), isolated diastolic elevation in 15 children (11.7%), and concurrent elevation of SBP and DBP in 34 children (26.6%). The distributions of SBP and DBP and the corresponding screening BP categories are presented in Table 1.

**Table 1:** Distribution of systolic and diastolic blood pressure and screening BP categories among preschool children (*N* = 128).

Blood Pressure	Statistic/Screening Classification	Total	
		*N*	Value
SBP [mmHg]	Mean ± SD	128	112.68 ± 12.21
	Median (Q1–Q3)	128	110.25 (104.88–119.00)
DBP [mmHg]	Mean ± SD	128	70.84 ± 10.81
	Median (Q1–Q3)	128	69.50 (63.38–76.13)
Screening BP	SBP and DBP < 95th percentile	54	42.2%
	Isolated SBP ≥ 95th percentile	25	19.5%
	Isolated DBP ≥ 95th percentile	15	11.7%
	Both SBP and DBP ≥ 95th percentile	34	26.6%
	SBP and/or DBP ≥ 95th percentile (total)	74	57.8%

No statistically significant differences in BMI, FAT, WHtR, or MVPA were observed between children with BP < 95th percentile and those with BP ≥ 95th percentile in the total sample (all *p* > 0.05). Similarly, sex-stratified analyses revealed no statistically significant between-group differences in any of the examined variables among girls or boys (all *p* > 0.05) (Table 2).

**Table 2:** Anthropometric indicators and moderate-to-vigorous physical activity according to the blood pressure threshold, overall and by sex.

Variable	BP Category	Total			Girls			Boys		
		*N*	Mean ± SD	*p*-Value	*N*	Mean ± SD	*p*-Value	*N*	Mean ± SD	*p*-Value
**BMI (kg/m^2^)**	<95th percentile	54	15.86 ± 2.45	0.9629	27	15.39 ± 1.78	0.1757	27	16.32 ± 2.93	0.3318
	≥95th percentile	74	15.88 ± 1.77		38	16.03 ± 1.95		36	15.71 ± 1.56	
**FAT (%)**	<95th percentile	54	19.18 ± 4.45	0.1919	27	19.43 ± 4.75	0.2763	27	18.94 ± 4.20	0.4893
	≥95th percentile	74	20.24 ± 4.56		38	20.81 ± 5.34		36	19.63 ± 3.54	
**WHtR**	<95th percentile	54	0.463 ± 0.032	0.9778	27	0.457 ± 0.034	0.9431	27	0.468 ± 0.028	1.0000
	≥95th percentile	74	0.463 ± 0.033		38	0.458 ± 0.032		36	0.468 ± 0.033	
**MVPA (min/day)**	<95th percentile	30	34.49 ± 20.46	0.9232	14	29.87 ± 12.66	0.5934	16	38.54 ± 25.16	0.9302
	≥95th percentile	51	34.96 ± 22.34		28	32.62 ± 20.23		23	37.82 ± 24.83	

BMI—body mass index; FAT—body fat percentage; WHtR—waist-to-height ratio; MVPA—moderate-to-vigorous physical activity; BP—blood pressure; SBP—systolic blood pressure; DBP—diastolic blood pressure; SD—standard deviation; *N*—number of participants. Values are presented as mean ± SD. Between-group comparisons were performed using Welch’s independent-samples *t*-test. Screening BP ≥ 95th percentile was defined as SBP and/or DBP at or above the age-, sex-, and height-specific 95th percentile according to the 2016 European Society of Hypertension guidelines. Statistical significance was set at *p* < 0.05.

Table 3 presents the distribution of BMI, body fat percentage (FAT), WHtR, and MVPA categories in the total sample and by sex. Most children had a BMI < 85th percentile (78.9%) and a WHtR < 0.50 (88.3%). For FAT, 58.6% of children were below the 85th percentile, whereas 41.4% had FAT ≥ 85th percentile. Only 13.6% of children with valid accelerometer data accumulated an average of ≥60 min of MVPA per day.

The distribution of FAT categories differed significantly by sex (Fisher’s exact test, *p* = 0.0193), whereas no significant sex differences were observed for BMI (*p* = 0.5194), WHtR (*p* = 0.0570), or MVPA (*p* = 0.1080). Percentages for girls and boys were calculated within each sex category.

No significant differences in SBP or DBP were observed between children with weekday screen time ≤ 1 h/day and >1 h/day, either in the total sample or in sex-stratified analyses (Table 4).

Children with weekend screen time > 1 h/day had higher SBP and DBP values than those with screen time ≤ 1 h/day. In the total sample, these differences did not reach statistical significance. In exploratory sex-stratified analyses, significant differences were observed among boys: SBP was 114.0 vs. 107.5 mmHg (*p* = 0.0101), and DBP was 73.0 vs. 65.0 mmHg (*p* = 0.0106) in the >1 h/day and ≤1 h/day groups, respectively (Table 5). No statistically significant differences were observed among girls.

In the total study population, SBP showed weak positive correlations with BMI (r = 0.34), FAT (r = 0.34), and weekend screen time (r = 0.19), whereas DBP showed weak positive correlations with FAT (r = 0.19) and weekend screen time (r = 0.18); all starred correlations in Table 6 were statistically significant. Among girls, SBP correlated moderately with BMI (r = 0.44) and weakly with FAT (r = 0.37), while DBP correlated weakly with FAT (r = 0.25). Among boys, SBP correlated weakly with FAT (r = 0.38) and weekend screen time (r = 0.34), and DBP correlated weakly with weekend screen time (r = 0.35). No statistically significant correlations were observed between MVPA and SBP or DBP in the total sample or in sex-stratified analyses.

In unadjusted logistic regression analyses, BMI ≥ 85th percentile was not significantly associated with screening BP ≥ 95th percentile (OR = 1.31; 95% CI: 0.55–3.15; *p* = 0.5425). Children with FAT ≥ 85th percentile had higher estimated odds of elevated screening BP (OR = 2.06; 95% CI: 0.99–4.29), although the association did not reach statistical significance (*p* = 0.0531). Weekend screen time > 1 h/day was significantly associated with higher odds of screening BP ≥ 95th percentile (OR = 2.29; 95% CI: 1.07–4.90; *p* = 0.0327). No statistically significant associations were observed for WHtR ≥ 0.50, MVPA < 60 min/day, or weekday screen time > 1 h/day (Table 7). Given the multiple comparisons performed, these findings should be interpreted cautiously.

The proportions of children with screening BP ≥ 95th percentile were 63.0% (17/27) for BMI ≥ 85th percentile versus 56.4% (57/101) for BMI < 85th percentile; 67.9% (36/53) for FAT ≥ 85th percentile versus 50.7% (38/75) for FAT < 85th percentile; and 60.0% (9/15) for WHtR ≥ 0.50 versus 57.5% (65/113) for WHtR < 0.50. For MVPA < 60 versus ≥60 min/day, the corresponding proportions were 61.4% (43/70) and 72.7% (8/11). For weekday screen time > 1 versus ≤ 1 h/day, they were 61.8% (21/34) and 56.8% (50/88), respectively; for weekend screen time, they were 65.4% (53/81) and 45.2% (19/42).

In multivariable logistic regression models adjusted for age, sex, weekday screen time, and weekend screen time, FAT ≥ 85th percentile was significantly associated with screening BP ≥ 95th percentile (aOR = 2.47; 95% CI: 1.09–5.59; *p* = 0.0304). In contrast, neither BMI ≥ 85th percentile (aOR = 1.55; 95% CI: 0.60–4.01; *p* = 0.3642) nor WHtR ≥ 0.50 (aOR = 1.58; 95% CI: 0.44–5.68; *p* = 0.4854) was significantly associated with screening BP ≥ 95th percentile. Weekend screen time > 1 h/day was also significantly associated with screening BP ≥ 95th percentile in all three models (aOR range: 2.39–2.57; all *p* < 0.05) (Table 8).

As the three adiposity indicators were examined in separate models, these findings should not be interpreted as evidence that FAT is superior to BMI or WHtR.

In the conventional multivariable logistic regression models, weekend screen time > 1 h/day was significantly associated with screening BP ≥ 95th percentile across all three models (aOR range: 2.39–2.57; all *p* < 0.05), whereas weekday screen time was not significantly associated with the outcome. These findings should be interpreted cautiously because of the exploratory nature of the analyses and the absence of adjustment for multiple comparisons.

Given the high prevalence of screening BP ≥ 95th percentile, modified Poisson regression with robust variance estimation was performed as an additional analysis. FAT ≥ 85th percentile was significantly associated with elevated screening BP (aPR = 1.41; 95% CI: 1.04–1.92; *p* = 0.0277), whereas BMI ≥ 85th percentile (aPR = 1.18; 95% CI: 0.85–1.64; *p* = 0.3322) and WHtR ≥ 0.50 (aPR = 1.17; 95% CI: 0.78–1.76; *p* = 0.4495) were not significantly associated with the outcome. These results were broadly consistent with the multivariable logistic regression findings.

As a sensitivity analysis, mixed-effects logistic regression models with a random intercept for kindergarten were fitted. The estimated random-intercept variance approached zero in all three models (ICC ≈ 0), and the fixed-effect odds ratios were virtually identical to those obtained from conventional logistic regression (BMI: OR = 1.55; FAT: OR = 2.47; WHtR: OR = 1.58). Although numerical optimization converged, the models yielded boundary solutions, limiting the interpretability of the random-effects component. Therefore, the conventional multivariable logistic regression models were retained as the primary analyses.

## 4. Discussion

The present study examined associations between objectively measured moderate-to-vigorous physical activity (MVPA), parent-reported screen time, adiposity indicators, and elevated screening blood pressure in children aged 5–6 years. Following verification of individual BP classifications, SBP and/or DBP ≥ 95th percentile was observed in 74 of 128 children (57.8%). FAT ≥ 85th percentile was significantly associated with elevated screening BP after adjustment for age, sex, and screen time (aOR = 2.47; 95% CI: 1.09–5.59), with a corresponding association observed in modified Poisson regression (aPR = 1.41; 95% CI: 1.04–1.92). However, the unadjusted association for FAT did not reach statistical significance, and neither BMI ≥ 85th percentile nor WHtR ≥ 0.50 was significantly associated with the outcome in the adjusted models. Weekend screen time > 1 h/day was also associated with elevated screening BP in the regression analyses, whereas no significant association was found for MVPA. These findings should be interpreted cautiously in view of the study design, multiple comparisons, and limitations of the measurements.

The high proportion of children with SBP and/or DBP ≥ 95th percentile (57.8%) requires careful interpretation. Although this proportion decreased following verification of individual BP classifications, it remained unusually high. Song et al. [23] estimated the global prevalence of hypertension among children and adolescents at approximately 4%; however, this estimate refers to a different outcome and population context and is not directly comparable with elevated BP values obtained during a single screening session.

Several methodological factors may have contributed to the high proportion observed in the present study, including the use of an oscillometric device without independently confirmed pediatric validation for this age group and the absence of auscultatory confirmation. Although the measurements followed a standardized protocol, potential measurement-related effects cannot be completely excluded. Furthermore, children’s age was recorded in completed years rather than exact months, which may have introduced some imprecision in the assignment of age-specific BP reference thresholds.

Importantly, the BP values were obtained during a single assessment session. Therefore, the observed proportion should not be interpreted as the prevalence of clinically diagnosed hypertension but rather as the proportion of children with elevated screening BP who require further evaluation and confirmation through repeated measurements at subsequent visits.

Among the adiposity indicators examined, FAT ≥ 85th percentile was significantly associated with elevated screening BP in the adjusted logistic regression model (aOR = 2.47; 95% CI: 1.09–5.59), whereas the unadjusted association did not reach statistical significance (OR = 2.06; 95% CI: 0.99–4.29; *p* = 0.0531). The association was also observed in the modified Poisson regression analysis (aPR = 1.41; 95% CI: 1.04–1.92), which provides a more interpretable measure of relative prevalence given the high frequency of elevated screening BP in the sample.

In contrast, BMI ≥ 85th percentile and WHtR ≥ 0.50 were not significantly associated with elevated screening BP in the adjusted models. However, the presence of statistical significance for FAT and its absence for BMI or WHtR should not be interpreted as evidence that FAT is a superior indicator. The adiposity measures were examined in separate models, and no formal statistical comparison of their associations or predictive performance was conducted.

These findings are consistent with previous research identifying excess adiposity as an important factor associated with elevated BP in childhood [5,24,25]. BMI remains a practical indicator for routine screening, whereas BIA-derived body fat percentage may provide complementary information. Nevertheless, differences between adiposity indicators should be interpreted cautiously, particularly given the limitations of BIA-based estimation and the use of reference curves developed in a different pediatric population.

No statistically significant association was observed between objectively measured MVPA and elevated screening BP in the present study. However, valid accelerometer data were available for only 81 of the 128 participants (63.3%), which may have reduced statistical power and introduced selection bias. Therefore, the absence of a statistically significant association should not be interpreted as evidence that physical activity is unrelated to BP in preschool-aged children.

An additional methodological consideration is the timing of the measurements. BP was assessed during a single session, whereas accelerometer monitoring began on the assessment day and continued over the subsequent seven-day period. Consequently, the recorded physical activity partly reflected behavior occurring after BP measurement, limiting the interpretation of the temporal relationship between MVPA and BP.

Previous research supports the broader importance of physical activity for cardiometabolic health in children. Ekelund et al. [7] reported favorable associations between higher MVPA and cardiometabolic risk profiles, while meta-analyses by Hassan et al. [8] and Tozo et al. [9] investigated the effects of physical activity interventions on BP. However, a systematic review and meta-analysis by García-Hermoso et al. [26], focusing specifically on preschool-aged children, found that exercise interventions improved physical fitness and body composition but did not significantly reduce BP. Nevertheless, further prospective studies using repeated BP assessments and objectively measured physical activity are needed to clarify these relationships in preschool-aged children.

In exploratory sex-stratified analyses, boys with weekend screen time >1 h/day had higher SBP and DBP values than boys with screen time ≤ 1 h/day. However, no formal sex-by-screen-time interaction was tested; therefore, statistically significant differences observed among boys but not among girls should not be interpreted as evidence of a sex-specific association.

In the revised regression analyses, weekend screen time > 1 h/day was significantly associated with screening BP ≥ 95th percentile in the unadjusted logistic regression model (OR = 2.29; 95% CI: 1.07–4.90) and in all three multivariable logistic regression models (aOR range: 2.39–2.57). In the additional modified Poisson regression analyses, the association remained statistically significant in Models A and C but not in Model B, indicating some sensitivity to model specification.

These findings are consistent with previous reports suggesting potential unfavorable associations between longer screen time and cardiovascular health in children [10,11]. Nevertheless, the screen time data were based on parental reports, and the exploratory analyses were conducted without adjustment for multiple comparisons. Therefore, these findings should be interpreted cautiously and require confirmation in larger prospective studies.

Overall, the findings suggest that BIA-derived body fat percentage may provide complementary information when examining associations between adiposity and elevated screening BP in preschool-aged children. However, the results do not establish the superiority of FAT over BMI or WHtR, nor do they justify replacing routinely used anthropometric indicators with BIA-based body fat estimates. The observed association between weekend screen time and elevated screening BP requires further investigation, particularly given the reliance on parental reports and the exploratory nature of the analyses. Similarly, the absence of a statistically significant association between MVPA and BP should be interpreted in the context of the reduced accelerometry sample. As BP was assessed during a single session, these findings should be regarded as exploratory and require confirmation in larger prospective studies.

### 4.1. Strengths

The main strengths of this study include the simultaneous assessment of several modifiable factors associated with elevated blood pressure in preschool-aged children, including objectively measured physical activity, screen time, and multiple indicators of adiposity. An important strength was the use of seven-day accelerometer-based monitoring of physical activity, which reduced the risk of bias associated with subjective assessment, together with a standardized blood pressure measurement protocol.

Another strength was the inclusion of several adiposity indicators, including BMI, body fat percentage, and WHtR, allowing their associations with elevated screening BP to be examined in separate statistical models. An additional value of the study is the focus on a relatively narrow age group of 5–6-year-old children, which remains underrepresented in research on early cardiovascular risk factors.

### 4.2. Limitations

This study has several methodological limitations. First, its cross-sectional design precludes causal inference. Furthermore, blood pressure was assessed during a single screening session; therefore, SBP and/or DBP values ≥ 95th percentile cannot be considered sufficient for a clinical diagnosis of hypertension. Additional limitations include the use of oscillometric blood pressure measurements without auscultatory confirmation and the lack of identified independent pediatric validation of the Omron HBP-1100 device in children aged 5–6 years. Furthermore, children’s ages were recorded in completed years rather than exact months, which may have introduced some imprecision in the assignment of age-specific BP reference thresholds.

Body fat percentage (FAT) was estimated using bioelectrical impedance analysis (BIA), the results of which may be influenced by the device used, the prediction algorithm, and measurement conditions, particularly participants’ hydration status. Although parents were instructed to ensure that their children fasted for at least 8 h before the assessment, variability in physiological conditions cannot be entirely excluded. Furthermore, the reference curves developed by McCarthy et al. were derived from a British population using a different BIA analyzer, which limits the direct comparability of the resulting FAT estimates. Moreover, BMI, FAT, and WHtR were examined in separate regression models without a formal comparison of their predictive performance; therefore, the results do not establish the superiority of any individual adiposity indicator.

Screen time was assessed using parental reports collected through a custom-developed, non-validated questionnaire. This approach may have introduced recall bias and inaccuracies in exposure estimates, particularly regarding screen use during kindergarten hours, the type of screen content viewed, and the simultaneous use of multiple devices. An additional limitation was the use of a single cutoff value for screen time classification.

Valid accelerometry data were obtained from 81 of the 128 participants, which may have reduced the statistical power of analyses involving moderate-to-vigorous physical activity (MVPA) and increased the risk of selection bias. The lack of complete temporal alignment between measurements should also be considered. Blood pressure was assessed during a single visit, whereas physical activity monitoring began on the same day and continued for seven consecutive days. Consequently, the recorded physical activity included the period following blood pressure assessment, limiting the ability to establish the temporal sequence of the observed associations.

Multiple statistical analyses were performed, including sex-stratified comparisons, without formal adjustment for multiple testing. This may have increased the risk of Type I error; therefore, findings from exploratory analyses should be interpreted with caution. In addition, the multivariable regression models included a limited set of available covariates, and residual confounding due to unmeasured or unaccounted-for factors cannot be ruled out.

Finally, although kindergartens were randomly selected from different geographical areas of Rzeszów, children’s participation was voluntary and restricted to public kindergarten settings. Detailed data enabling comparisons between participating and non-participating children were unavailable, potentially limiting the representativeness of the study sample and the generalizability of the findings to the broader population of preschool-aged children.

## 5. Conclusions

The present study demonstrated that elevated body fat percentage (FAT ≥ 85th percentile) was significantly associated with screening BP ≥ 95th percentile among children aged 5–6 years after adjustment for age, sex, and screen time (aOR = 2.47; 95% CI: 1.09–5.59). This association was also supported by modified Poisson regression (aPR = 1.41; 95% CI: 1.04–1.92). In contrast, BMI ≥ 85th percentile and WHtR ≥ 0.50 were not significantly associated with elevated screening BP in the adjusted models. However, these findings do not establish the superiority of FAT over other adiposity indicators, as no formal comparison of their predictive performance was performed. Weekend screen time > 1 h/day was also associated with elevated screening BP in the revised regression analyses. Exploratory sex-stratified comparisons indicated higher SBP and DBP values among boys with longer weekend screen time; however, these findings should not be interpreted as evidence of a sex-specific association. No statistically significant association between objectively measured MVPA and screening BP was observed, although the reduced accelerometry sample limits the interpretation of this result.

Overall, the findings suggest that BIA-derived body fat percentage may provide complementary information when assessing adiposity-related associations with screening BP in preschool-aged children, without replacing established anthropometric indicators. Importantly, the unusually high proportion of children with SBP and/or DBP ≥ 95th percentile (57.8%) was based on a single screening session and should not be interpreted as the prevalence of clinically diagnosed hypertension. Further prospective studies involving larger samples, repeated BP measurements, and standardized assessments of physical activity, screen time, and body composition are needed to confirm these associations.

## Figures and Tables

**Table 3:** Distribution of anthropometric indicators and moderate-to-vigorous physical activity categories by sex.

Variables	Cut-Off Points	Classification	Total *N* = 128 (81 MVPA)		Girls *N* = 65; 50.8%		Boys *N* = 63; 49.2%		*p*-Value
			*N*; %	Me ± QD	*N*; %	Me ± QD	*N*; %	Me ± QD	
BMI [kg/m^2^]	<85th percentile	Below cut-off	101; 78.9%	15.00 ± 0.70	53; 81.5%	15.10 ± 0.70	48; 76.2%	15.00 ± 0.74	0.5194
	≥85th percentile	At/above cut-off	27; 21.1%	17.60 ± 0.85	12; 18.5%	17.85 ± 0.64	15; 23.8%	17.50 ± 1.10	—
FAT [%]	<85th percentile	Below cut-off	75; 58.6%	17.60 ± 1.80	45; 69.2%	18.50 ± 2.30	30; 47.6%	17.10 ± 1.00	0.0193 *
	≥85th percentile	At/above cut-off	53; 41.4%	22.70 ± 2.20	20; 30.8%	24.70 ± 1.38	33; 52.4%	20.70 ± 1.40	—
WHtR [unitless]	<0.50	Within cut-off	113; 88.3%	0.459 ± 0.015	61; 93.8%	0.455 ± 0.016	52; 82.5%	0.461 ± 0.015	0.0570
	≥0.50	Above cut-off	15; 11.7%	0.520 ± 0.010	4; 6.2%	0.531 ± 0.016	11; 17.5%	0.513 ± 0.007	—
MVPA [min]	≥60 min	Meets recommendation	11; 13.6%	68.86 ± 4.82	3; 7.1%	68.00 ± 2.79	8; 20.5%	70.50 ± 6.96	0.1080
	<60 min	Below recommendation	70; 86.4%	28.79 ± 12.16	39; 92.9%	29.14 ± 9.79	31; 79.5%	28.43 ± 14.04	—

BMI—Body Mass Index; FAT—Body Fat; WHtR—Waist-to-Height Ratio; MVPA—Moderate-to-Vigorous Physical Activity; *N*—Number of participants; Me—Median; QD—Quartile Deviation; * *p* < 0.05. BMI and FAT were classified using the 85th-percentile threshold; WHtR using a cut-off of 0.50 (<0.50 vs. ≥0.50); and MVPA using an average of 60 min/day. Percentages for girls and boys are calculated within sex. One *p*-value is reported for each dichotomous variable and was calculated using Fisher’s exact test.

**Table 4:** Comparison of systolic and diastolic blood pressure according to weekday screen time: overall and by sex.

Blood Pressure	Weekday Screen Time Classification [Cut-Off Points]	Total			Girls			Boys		
		*N*	Me ± QD	*p*-Value	*N*	Me ± QD	*p*-Value	*N*	Me ± QD	*p*-Value
SBP [mmHg]	≤1 h/day	88	109.2 ± 7.1	0.3593	43	110.0 ± 9.7	0.7733	45	109.0 ± 4.7	0.2357
	>1 h/day	34	114.2 ± 9.0		20	112.5 ± 10.7		14	115.0 ± 5.7	
DBP [mmHg]	≤1 h/day	88	69.5 ± 6.6	0.1660	43	67.5 ± 6.5	0.3333	45	70.0 ± 7.0	0.2690
	>1 h/day	34	69.0 ± 7.5		20	68.2 ± 6.2		14	72.7 ± 9.5	

SBP—Systolic Blood Pressure; DBP—Diastolic Blood Pressure; *N*—Number of participants; Me—Median; QD—Quartile Deviation. Weekday screen time was categorized as ≤1 h/day or >1 h/day using the threshold summarized by McArthur et al. [22]. *p*-values were calculated using the Mann–Whitney U test; *p* < 0.05.

**Table 5:** Comparison of systolic and diastolic blood pressure according to weekend screen time, overall and by sex.

Blood Pressure	Weekend Screen Time Classification [Cut-Off Points]	Total			Girls			Boys		
		*N*	Me ± QD	*p*-Value	*N*	Me ± QD	*p*-Value	*N*	Me ± QD	*p*-Value
SBP [mmHg]	≤1 h/day	42	107.75 ± 4.25	0.0863	19	108.00 ± 9.75	0.9641	23	107.50 ± 3.25	0.0101 *
	>1 h/day	81	113.00 ± 8.50		44	111.75 ± 10.62		37	114.00 ± 6.25	
DBP [mmHg]	≤1 h/day	42	67.00 ± 6.00	0.0748	19	71.50 ± 7.00	0.9462	23	65.00 ± 5.00	0.0106 *
	>1 h/day	81	69.50 ± 6.00		44	67.50 ± 5.37		37	73.00 ± 6.75	

SBP—Systolic Blood Pressure; DBP—Diastolic Blood Pressure; *N*—Number of participants; Me—Median; QD—Quartile Deviation. Weekend screen time was categorized as ≤1 h/day or >1 h/day using the threshold summarized by McArthur et al. [22]. *p*-values were calculated using the Mann–Whitney U test; * *p* < 0.05.

**Table 6:** Spearman’s rank correlations between anthropometric indicators, physical activity, screen time, and systolic and diastolic blood pressure, overall and by sex.

Group	Variable	*N*	SBP [r]	DBP [r]
Girls	BMI	65	0.44 *	0.20
Girls	FAT	65	0.37 *	0.25 *
Girls	WHtR	65	0.01	0.08
Girls	MVPA	42	0.20	0.02
Girls	Weekday screen time	63	0.11	0.21
Girls	Weekend screen time	63	0.07	0.05
Boys	BMI	63	0.23	0.04
Boys	FAT	63	0.38 *	0.15
Boys	WHtR	63	0.11	0.11
Boys	MVPA	39	0.02	−0.01
Boys	Weekday screen time	59	0.09	0.07
Boys	Weekend screen time	60	0.34 *	0.35 *
Total	BMI	128	0.34 *	0.12
Total	FAT	128	0.34 *	0.19 *
Total	WHtR	128	0.07	0.08
Total	MVPA	81	0.08	−0.01
Total	Weekday screen time	122	0.10	0.14
Total	Weekend screen time	123	0.19 *	0.18 *

BMI—body mass index; FAT—body fat percentage; WHtR—waist-to-height ratio; MVPA—moderate-to-vigorous physical activity; SBP—systolic blood pressure; DBP—diastolic blood pressure; *N*—number of valid paired observations; r—Spearman’s correlation coefficient. * *p* < 0.05.

**Table 7:** Associations of selected factors with BP ≥ 95th percentile.

Variables	OR (95% CI)	*p*-Value
BMI	1.31 (0.55–3.15)	0.5425
FAT	2.06 (0.99–4.29)	0.0531
WHtR	1.11 (0.37–3.32)	0.8552
MVPA	0.60 (0.15–2.44)	0.4741
Weekday screen time	1.23 (0.55–2.76)	0.6197
Weekend screen time	2.29 (1.07–4.90)	0.0327

BMI—Body Mass Index; FAT—Body Fat Percentage; WHtR—Waist-to-Height Ratio; MVPA—Moderate-to-Vigorous Physical Activity; OR—Odds Ratio; CI—Confidence Interval; *p* < 0.05. Reference categories: BMI < 85th percentile, FAT < 85th percentile, WHtR <0.50, MVPA ≥ 60 min/day, weekday screen time ≤ 1 h/day, and weekend screen time ≤ 1 h/day. Outcome: SBP and/or DBP ≥ 95th percentile vs. both SBP and DBP < 95th percentile.

**Table 8:** Multivariable logistic regression models for BP ≥ 95th percentile.

Model/Variable	aOR	95% CI	*p*-Value	*N*
Model A: BMI ≥ 85th percentile	1.55	0.60–4.01	0.3642	122
Sex (boys vs. girls)	1.01	0.48–2.13	0.9828	122
Age (6 vs. 5 years)	0.87	0.41–1.83	0.7090	122
Weekday screen time (>1 vs. ≤1 h/day)	0.85	0.35–2.07	0.7170	122
Weekend screen time (>1 vs. ≤1 h/day)	2.53	1.09–5.89	0.0310	122
Model B: FAT ≥ 85th percentile	2.47	1.09–5.59	0.0304	122
Sex (boys vs. girls)	0.83	0.38–1.81	0.6322	122
Age (6 vs. 5 years)	1.04	0.48–2.28	0.9148	122
Weekday screen time (>1 vs. ≤1 h/day)	0.80	0.32–2.00	0.6374	122
Weekend screen time (>1 vs. ≤1 h/day)	2.39	1.01–5.65	0.0464	122
Model C: WHtR ≥ 0.50	1.58	0.44–5.68	0.4854	122
Sex (boys vs. girls)	0.98	0.46–2.07	0.9495	122
Age (6 vs. 5 years)	0.88	0.42–1.87	0.7486	122
Weekday screen time (>1 vs. ≤1 h/day)	0.83	0.34–2.04	0.6883	122
Weekend screen time (>1 vs. ≤1 h/day)	2.57	1.11–5.96	0.0280	122

aOR—adjusted odds ratio; CI—confidence interval. All three models were adjusted for weekday screen time (>1 vs. ≤1 h/day) and weekend screen time (>1 vs. ≤1 h/day), in addition to age and sex. BMI, FAT, and WHtR were entered in separate models to avoid simultaneous inclusion of related adiposity measures.

## Data Availability

The data presented in this study are available from the corresponding author upon reasonable request, subject to ethical restrictions and the protection of participants’ confidentiality.

## Abbreviations

The following abbreviations are used in this manuscript:

SBP	Systolic Blood Pressure
DBP	Diastolic Blood Pressure
BMI	Body Mass Index
FAT	Body Fat Percentage
WHtR	Waist-to-Height Ratio
MVPA	Moderate-to-Vigorous Physical Activity
ESH	European Society of Hypertension
WHO	World Health Organization
OR	Odds Ratio
aOR	Adjusted Odds Ratio
CI	Confidence Interval
ICC	Intraclass Correlation Coefficient
ISAK	International Society for the Advancement of Kinanthropometry
Me	Median
QD	Quartile Deviation
BP	Blood Pressure
BIA	Bioelectrical Impedance Analysis
PR	Prevalence Ratio
aPR	Adjusted Prevalence Ratio
SD	Standard Deviation
Q1	First Quartile
Q3	Third Quartile

## References

[1] Yu Y.-L., Renaers E., Martens D.S., Sleurs H., Massot A., Berden D., Verheyen L., Alfano R., Zhang D.-Y., An D.-W. (2026). Blood Pressure Trajectory From Birth to Preschool and School Age in the ENVIRONAGE Birth Cohort. JAMA Netw. Open.

[2] Fujiwara T., Kaneko H., Hayashi K., Azegami T., Mizuno A., Yano Y. (2026). Hypertension in Children and Adolescents: Emerging Global Evidence and Clinical Implications. Hypertens. Res..

[3] Martín-Espinosa N., Díez-Fernández A., Sánchez-López M., Rivero-Merino I., Lucas-De La Cruz L., Solera-Martínez M., Martínez-Vizcaíno V. (2017). Movi-Kids Group Prevalence of High Blood Pressure and Association with Obesity in Spanish Schoolchildren Aged 4–6 Years Old. PLoS ONE.

[4] Flynn J.T., Kaelber D.C., Baker-Smith C.M., Blowey D., Carroll A.E., Daniels S.R., de Ferranti S.D., Dionne J.M., Falkner B., Flinn S.K. (2017). Clinical Practice Guideline for Screening and Management of High Blood Pressure in Children and Adolescents. Pediatrics.

[5] Jeong S.I., Kim S.H. (2024). Obesity and Hypertension in Children and Adolescents. Clin. Hypertens..

[6] Tang X., Liu Y., Hu J., Zhai L., Jia L., Ding N., Ma Y., Wen D. (2022). Association of Waist Circumference with Blood Pressure and Familial Dietary Habits in Preschool Children: A Cross-Sectional Study in Northeastern China. Ital. J. Pediatr..

[7] Ekelund U., Luan J., Sherar L.B., Esliger D.W., Griew P., Cooper A. (2012). International Children’s Accelerometry Database (ICAD) Collaborators, for the Moderate to Vigorous Physical Activity and Sedentary Time and Cardiometabolic Risk Factors in Children and Adolescents. JAMA.

[8] Hassan M.A., Zhou W., Ye M., He H., Gao Z. (2024). The Effectiveness of Physical Activity Interventions on Blood Pressure in Children and Adolescents: A Systematic Review and Network Meta-Analysis. J. Sport Health Sci..

[9] Tozo J.V.A., Tadiotto M.C., Tozo T.A.A., de Menezes-Junior F.J., Mota J., de Pereira B.O., Rosário R., Leite N. (2025). Effects of Different Physical Exercise Programs on Blood Pressure in Overweight Children and Adolescents: Systematic Review and Meta-Analysis. BMC Pediatr..

[10] Farhangi M.A., Fathi Azar E., Manzouri A., Rashnoo F., Shakarami A. (2023). Prolonged Screen Watching Behavior Is Associated with High Blood Pressure among Children and Adolescents: A Systematic Review and Dose–Response Meta-Analysis. J. Health Popul. Nutr..

[11] Horner D., Jahn M., Bønnelykke K., Chawes B., Flensborg-Madsen T., Schoos A.M., Stokholm J., Rasmussen M.A. (2025). Screen Time Is Associated With Cardiometabolic and Cardiovascular Disease Risk in Childhood and Adolescence. J. Am. Heart Assoc..

[12] Lurbe E., Agabiti-Rosei E., Cruickshank J., Dominiczak A., Erdine S., Hirth A., Invitti C., Litwin M., Mancia G., Pall D. (2016). 2016 European Society of Hypertension Guidelines for the Management of High Blood Pressure in Children and Adolescents. J. Hypertens..

[13] Kułaga Z., Grajda A., Gurzkowska B., Góźdź M., Wojtyło M., Swiąder A., Różdżyńska-Świątkowska A., Litwin M. (2013). Polish 2012 Growth References for Preschool Children. Eur. J. Pediatr..

[14] McCarthy H.D., Cole T.J., Fry T., Jebb S.A., Prentice A.M. (2006). Body fat reference curves for children. Int. J. Obes..

[15] Brønd J.C., Andersen L.B., Arvidsson D. (2017). Generating ActiGraph Counts from Raw Acceleration Recorded by an Alternative Monitor. Med. Sci. Sports Exerc..

[16] Hinkley T., O’Connell E., Okely A.D., Crawford D., Hesketh K., Salmon J. (2012). Assessing Volume of Accelerometry Data for Reliability in Preschool Children. Med. Sci. Sports Exerc..

[17] Evenson K.R., Catellier D.J., Gill K., Ondrak K.S., McMurray R.G. (2008). Calibration of Two Objective Measures of Physical Activity for Children. J. Sports Sci..

[18] Bull F.C., Al-Ansari S.S., Biddle S., Borodulin K., Buman M.P., Cardon G., Carty C., Chaput J.-P., Chastin S., Chou R. (2020). World Health Organization 2020 Guidelines on Physical Activity and Sedentary Behaviour. Br. J. Sports Med..

[19] Choi L., Liu Z., Matthews C.E., Buchowski M.S. (2011). Validation of Accelerometer Wear and Nonwear Time Classification Algorithm. Med. Sci. Sports Exerc..

[20] Sadeh A., Sharkey K.M., Carskadon M.A. (1994). Activity-Based Sleep-Wake Identification: An Empirical Test of Methodological Issues. Sleep.

[21] Matłosz P., Wyszyńska J., Asif M., Szybisty A., Aslam M., Mazur A., Herbert J. (2021). Prevalence of Overweight, Obesity, Abdominal Obesity, and Obesity-Related Risk Factors in Polish Preschool Children: A Cross-Sectional Study. J. Clin. Med..

[22] McArthur B.A., Volkova V., Tomopoulos S., Madigan S. (2022). Global Prevalence of Meeting Screen Time Guidelines Among Children 5 Years and Younger: A Systematic Review and Meta-Analysis. JAMA Pediatr..

[23] Song P., Zhang Y., Yu J., Zha M., Zhu Y., Rahimi K., Rudan I. (2019). Global Prevalence of Hypertension in Children: A Systematic Review and Meta-Analysis. JAMA Pediatr..

[24] Moll J.C., Bohlken J., Kostev K. (2025). Prevalence of and Factors Associated with Hypertension in Children and Adolescents as Observed by German Pediatricians—A Case-Control Study. Children.

[25] Pool L.R., Aguayo L., Brzezinski M., Perak A.M., Davis M.M., Greenland P., Hou L., Marino B.S., Van Horn L., Wakschlag L. (2021). Childhood Risk Factors and Adulthood Cardiovascular Disease: A Systematic Review. J. Pediatr..

[26] García-Hermoso A., Alonso-Martinez A.M., Ramírez-Vélez R., Izquierdo M. (2020). Effects of Exercise Intervention on Health-Related Physical Fitness and Blood Pressure in Preschool Children: A Systematic Review and Meta-Analysis of Randomized Controlled Trials. Sports Med..

